# Validation and psychometric properties of the community assessment inventory in Iranian persons who use drug

**DOI:** 10.1186/s13011-020-00290-6

**Published:** 2020-07-18

**Authors:** Nader Salari, Amir Jalali, Behzad Abdam, Alireza Abdi, Hooman Daryoushi

**Affiliations:** 1 Department of Biostatistics, School of Public Health, Kermanshah University of Medical Sciences, Kermanshah, Iran; 2 Substance Abuse Prevention Research Center, Research Institute for Health, Kermanshah University of Medical Sciences, Kermanshah, Iran; 3 Student Research Committee, School of Nursing and Midwifery, Kermanshah University of Medical Sciences, Kermanshah, Iran; 4 Department of Nursing, School of Nursing and Midwifery, Kermanshah University of Medical Sciences, Kermanshah, Iran; 5 Department of Pediatrics, School of Medicine, Kermanshah University of Medical Sciences, Kermanshah, Iran

**Keywords:** Validity, Reliability, Psychometric, CAI, Iranian, PWUD

## Abstract

**Objective:**

Social support is a key factor in the treatment and rehabilitation process of persons who use drug (PWUD). This highlights the need for a valid and reliable tool for assessing social support. The cultural and psychometric properties of community assessment inventory (CAI) in PWUDs under methadone therapy were examined in Iran in 2019.

**Methods:**

The study was carried out as a validation and methodological study. At first, the original tool was translated into Farsi using forward-backward method. After ensuring face validity and content validity, construct reliability of the tool was supported using explorative and confirmatory factor analysis (EFA & CFA) using a sample group of 392 participants. The participants were selected through convenient-quota sampling from 24 drug clinics. Reliability of the questionnaire was supported using Pearson correlation coefficient and internal consistency based on Cronbach’s alpha.

**Results:**

To determine content validity of the tool, CVI and CVR of it were obtained, which were on average equal to 0.79 and 0.59 respectively. The EFA supported correlation of the 37itmes of the tool (KMO = 0.975, Chi-square = 15,051.6, P_value=_0.0001). The main indices of the model, based on CFA were higher than 0.9, which support goodness of fit of the model (χ2/DF = 2.98, CFI = 0.91, NFI, TLI = 0.905 GF = 0.92, REMSEA = 0.07, R2 = 0.99). Reliability of the tool based on internal consistency (Cronbach’s alpha) for the subscales were in 0.8–0.95 interval and equal to 0.85 for the whole tool.

**Conclusion:**

As the results showed, CAI had acceptable indices for Iranian PWUDs under methadone therapy. The tool can be used for assessing social support level in the study population. It is a reliable and valid tool for studies in pertinent fields.

## Introduction

Drug use disorders is a chronic, continues, and frequent behavior that creates physical and mental dependence in the individual [1]. In general, according to available statistics, 0.9% of the world’s population suffers from drug use disorders. Drug use disorders, that do not include alcohol, have troubled between 0.4 and 3.5% of the world population and 2% of Iranian population [2].

A persons who use drug (PWUD) tends to have major psychosocial needs that require a comprehensive and organized care to ensure treatment and prevent relapse [3]. Family and social supports in every aspect throughout the treatment and rehabilitation process can be a key factor to prevent relapse [4]. Researches have shown that challenges like undesirable social-economic condition [3, 5], poor family support [6], inadequate perceived social support [4], inter-personal conflicts, stigmatization and discrimination are normally experienced by PWUD care-seekers under treatment [4, 7].

Social support is a situation in which a person considers themself a member of a group or community that is well understood by individuals and supported from various physical, psychological, emotional, social and financial aspects [4]. Social supports attenuate stress, improve self-efficacy [8] and self-esteem, improve social, mental, and physical condition of the individual, and improve performance [9]. Perceived social support can create positive changes in the life of care-seekers and improve their social interactions [8, 9]. Such interactions boost one’s self-esteem and self-efficacy and make them more persistent to continue the rehabilitation process [10]. Social support can be an important factor in continuing treatment as well as preventing relapse in PWUD_s_ [7]. Social support can help them in the difficult process of rehabilitation by creating a sense of empathy and acceptance [11].

There are general tools to measure social support, which are used for PWUD_s_ as well [4, 7, 12]. The Community Assessment Inventory (CAI) is a social support assessment tool designed by Brown et al. (2004) for PWUDs. The tool is specifically used to measure social support in these care-seekers. It contains 37 statements and four subscales namely household, family, friends, and community [13]. Given the statements used in the tool and the fact that it is specifically designed for PWUD, CAI can be a good tool for PWUDs in Iran.

Taking into account the importance of assessing social support in PWUDs and absence of a specially designed, reliable, and normalized tool for this population, the present study is an attempt to examine validity and psychometric characteristics of the tool for Iranian PWUDs under methadone therapy.

## Methods

### Setting

The study was carried out as a validation and methodological study [14]. Cultural and psychometric characteristics of CAI in Iranian PWUDs under methadone therapy were examined. The study was conducted between September 2018 and July 2019 in Kermanshah – west of Iran.

### Participants

To determine face validity of the tool, it was provided to 20 PWUD_s_ in Kermanshah-based drug clinics. With regard to content validity, the tool was provided to 12 faculty board members and researchers. In addition, for explorative factor analysis (EFA) and confirmatory factor analysis (CFA), the tool was provided to 400 PWUDs under methadone therapy [13] from 24 Kermanshah-based drug clinics. The participants were selected through convenient-quota sampling. The demographics of participants are listed in Table 1 (seven questionnaires were omitted for being not completely filled out).

**Table 1:** Demographic characteristics of the study participants

Variable		N (%)
Gender	Male	376 (95.5)
	Female	16 (4.1)
Marital status	Single	264 (67.3)
	Married	128 (32.7)
Educational level	High School	172 (43.9)
	High school diploma	168 (42.9)
	Higher Education	52 (13.3)
Domicile	Urban	272 (69.4)
	Rural area	120 (30.6)
Job	Manual worker	88 (22.4)
	Freelance job	112 (28.6)
	Employed	184 (49)
Income/ monthly	Less than 100$	96 (24.5)
	100–300&	160 (40.8)
	More than 300$	136 (34.7)
Drug Use duration	Less than one year	88 (22.4)
	1–3 years	107 (27.3)
	3–5 years	138 (35.2)
	More than five years	59 (15.1)
Kind of drugs use	Opiate	204 (52)
	Heroin	124 (31.6)
	Asian Crack	28 (7.1)
	Tramadol	36 (9.2)
History of drugs treatment	Yes	236 (60.2)
	No	156 (39.8)
Numbers of Drugs Treatment	None	144 (36.7)
	1–3	156 (39.8)
	More than three	92 (23.5)
Relapse History	Yes	240 (61.2)
	No	152 (38.8)

Inclusion criteria were drug use disorder for at least one year, under methadone therapy for at least six months, desire to participate, not using synthetic drugs, and no physical and mental disease (medical file). Questionnaires that were not fully completed (less than 80% answered) were omitted.

### Community assessment inventory (CAI)

The CAI was designed in 2004 by Brown et al. and developed for PWUD_s_. The questionnaire measures support in four fields of household (six items), family (10 items), friends (eight items), and community (13 items). The questionnaire contains 37 items designed based on Likert’s four-point scale (1 = completely disagree, 2 = disagree, 3 = agree, 4 = completely agree). The higher the score, the higher the performance in any of the sub-scales [10].

### Cultural validation

After securing required permission for the designer of the tool, it was translated based on Wild et al.’s [15] ten steps following the translation and cultural comparability guideline. The ten steps are as follows:

- Communicating with the designer and securing the required permissions.
- Forward translation; two independent competent translators translated the tool from English into Farsi at the same time.
- The two translations were examined and compared and one version was extracted.
- Backward translation; the Farsi version was translated into English by two other independent and competent translators. They were asked to translate the meaning rather than word-by-work translation while remaining loyal to the origin.
- The researchers and experts examined the two translations and one version was extracted.
- The English translation was sent to the designer for feedbacks. Based on the feedbacks and considering the designer’s opinion, the original version of the tool was translated into Farsi.
- To ensure qualitative face validity, the tool was provided to 20 drug users under methadone therapy with minimum education level of high school. As to qualitative content validity, the tool was provided to 20 researcher and experts for their opinions (12 returned).
- The opinions were examined and implemented. None of the statements were removed at this stage.
- The tool was revised in terms of Farsi grammar and writing style by the research team.
- The final Farsi version was obtained.

After ensuring content validity through qualitative method, content validity through quantitative method was conducted as a supplementary method. To this end, CVR and CVI were computed (Table 2).

**Table 2:** The ratio and index of content validity and T-value of the tool items

No	CVR^a^	CVI^b^	Mean (SD)	Kurtosis^c^	Skewness^d^	T (cr)^e^	λ ^f^
1	0.67	0.92	3.55 (1.18)	− 2.278	− 0.255	− 3.918	0.393^**^
2	0.83	0.92	3.5 (1/18)	−1.595	−0.195	−4.072	0.365^**^
3	0.83	0.83	3.53 (1.16)	−2.013	− 0.247	−3.801	0.544^**^
4	0.83	0.92	3.52 (1.18)	− 1.909	− 0.234	−3.988	0.429^**^
5	0.67	0.92	3.53 (1.11)	−1.355	0.027	−5.523	0.427^**^
6	0.83	0.83	3.58 (1.1)	−1.298	−0.054	−5.299	0.311^**^
7	0.5	0.83	3.6 (1.23)	−2.794	−0.324	−4.431	0.543^**^
8	0.67	0.83	3.59 (1.21)	−2.77	−0.339	−4.325	0.404^**^
9	0.83	0.83	3.64 (1.22)	−3.087	−0.378	−4.279	0.348^**^
10	0.67	0.92	3.58 (1.23)	−2.6	−0.318	−4.506	0.388^**^
11	0.83	0.92	3.62 (1.17)	− 3.506	−0.429	−3.384	0.654^**^
12	0.5	0.83	3.58 (1.15)	−2.578	−0.316	−4.636	0.429^**^
13	0.67	0.75	3.57 (1.18)	−2.355	−0.288	−3.679	0.331^**^
14	0.83	0.83	3.56 (1.17)	−2.428	−0.297	−3.405	0.684^**^
15	0.67	0.83	3.55 (1.17)	−2.106	−0.258	−3.926	0.354^**^
16	0.67	0.92	3.6 (1.19)	−2.595	−0.318	−3.668	0.57^**^
17	0.83	0.92	3.48 (1.17)	−1.443	−0.177	−4.045	0.336^**^
18	0.67	0.83	3.59 (1.19)	−2.382	−0.292	−3.936	0.362^**^
19	0.83	0.75	3.56 (1.16)	−2.398	−0.294	−3.63	0.521^**^
20	0.67	0.83	3.56 (1.16)	−1.748	−0.214	−3.858	0.457^**^
21	0.67	0.92	3.5 (1.18)	−1.553	−0.19	−4.129	0.492^**^
22	0.5	0.83	3.53 (1.18)	−1.887	−0.231	−4.007	0.442^**^
23	0.5	0.92	3.57 (1.17)	−2.322	0.284	−3.81	0.489^**^
24	0.67	0.83	3.56 (1.18)	−2.146	−0.263	−3.983	0.501^**^
25	0.5	0.92	3.51 (1.12)	−1.152	−0.234	−1.152	0.512^**^
26	0.67	0.92	3.63 (1.21)	−3.44	−0.422	−0.934	0.558^**^
27	0.67	0.83	3.35 (1.22)	−0.408	−0.408	−1.005	0.539^**^
28	0.83	0.92	3.63 (1.21)	− 0.392	−0.392	−0.989	0.427^**^
29	0.83	0.92	3.53 (1.24)	−0.235	−0.235	−1.207	0.435^**^
30	0.83	0.83	3.52 (1.23)	−0.23	−0.23	−1.184	0.636^**^
31	0.83	0.92	3.63 (1.2)	− 0.404	−0.404	−0.962	0.579^**^
32	0.67	0.92	3.6 (1.2)	− 0.368	−0.368	−0.976	0.556^**^
33	0.67	0.83	3.63 (1.22)	−0.387	−0.387	−1.022	0.571^**^
34	0.5	0.75	3.57 (1.2)	−0.275	−0.275	−1.211	0.544^**^
35	0.5	0.92	3.62 (1.2)	− 0.389	−0.389	−0.97	0.523^**^
36	0.67	0.92	3.58 (1.2)	− 0.436	−0.436	−0.917	0.682^**^
37	0.83	0.92	3.61 (1.21)	− 0.435	−0.435	−0.98	0.6

** P_value < 0.001_

^a^ Content Validity Ratio, ^b^ Content Validity Index, ^c^ Kurtosis is a measure of whether the data are heavy-tailed or light-tailed relative to a normal distribution ^d^ Skewness is a measure of symmetry, or more precisely, the lack of symmetry, ^e^ The calculated values of t for all factor loadings of the first and second order are greater than 1.96 and are therefore significant at the 95% confidence level, ^f^ The specific value, which is denoted by the Lamda coefficient and the statistical symbol λ, is calculated from the sum of the factors of the factor loads related to all the variables of that factor

Construct validity was determined using EFA in SPSS (v. 25) and CFA was obtained in Lisrel (v. 8). Reliability of the scale was obtained through internal consistency method. Twenty PWUDs under methadone therapy filled the tool twice with two weeks internal. Afterwards, the obtained scores were compared using intra-class correlation. Cronbach’s alpha was used to determine internal consistency of the tool based on the subscales.

## Results

**Fig. 1:**
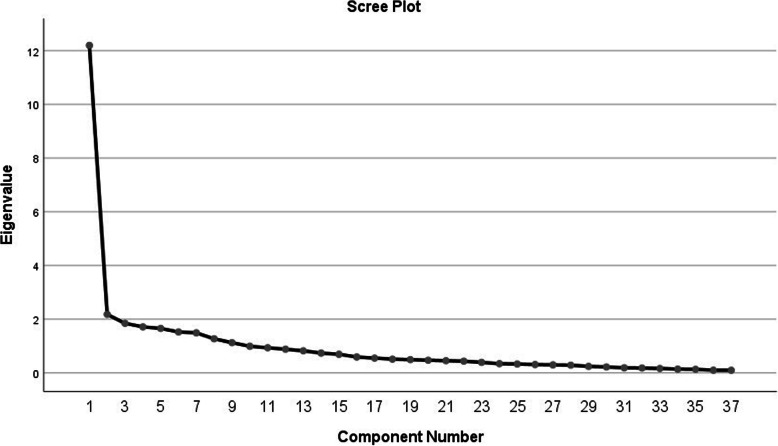
Scree plot of the extracted elements of the questionnaire

Before performing EFA, adequacy of sampling test was conducted to ensure that the sample size is large enough. The KMO test was obtained equal to 0.97 and confirmed the adequacy of the sample size for EFA. The Bartlett’s test of sphericity was equal to 15.51.6, which confirmed the adequacy of correlation between the scale items for factor analysis (*p*-value < 0.001). Given that H0 is not supported, a significant relationship between the variables is supported. Therefore, the presumptions of CFA were met and it was conducted on the answers by the subjects to the 37 statements of the scale. Varimax perpendicular rotation and principle component (PC) analysis were used. None of the statements were removed (Fig. 1).

### Construct validity – Confirmatory Factor Analysis

The mean score of statements ranged from 2.8 to five and the t-value ranged from 1.01–5.01. Skewness and Kurtosis were also at (− 2, 2) range. Therefore, normal distribution of the data is supported. In addition, the statements were at a desirable range given the factor load of each statement (*p* < 0.001), mean score, and t-value of the statements (Table 2).

**Fig. 2:**
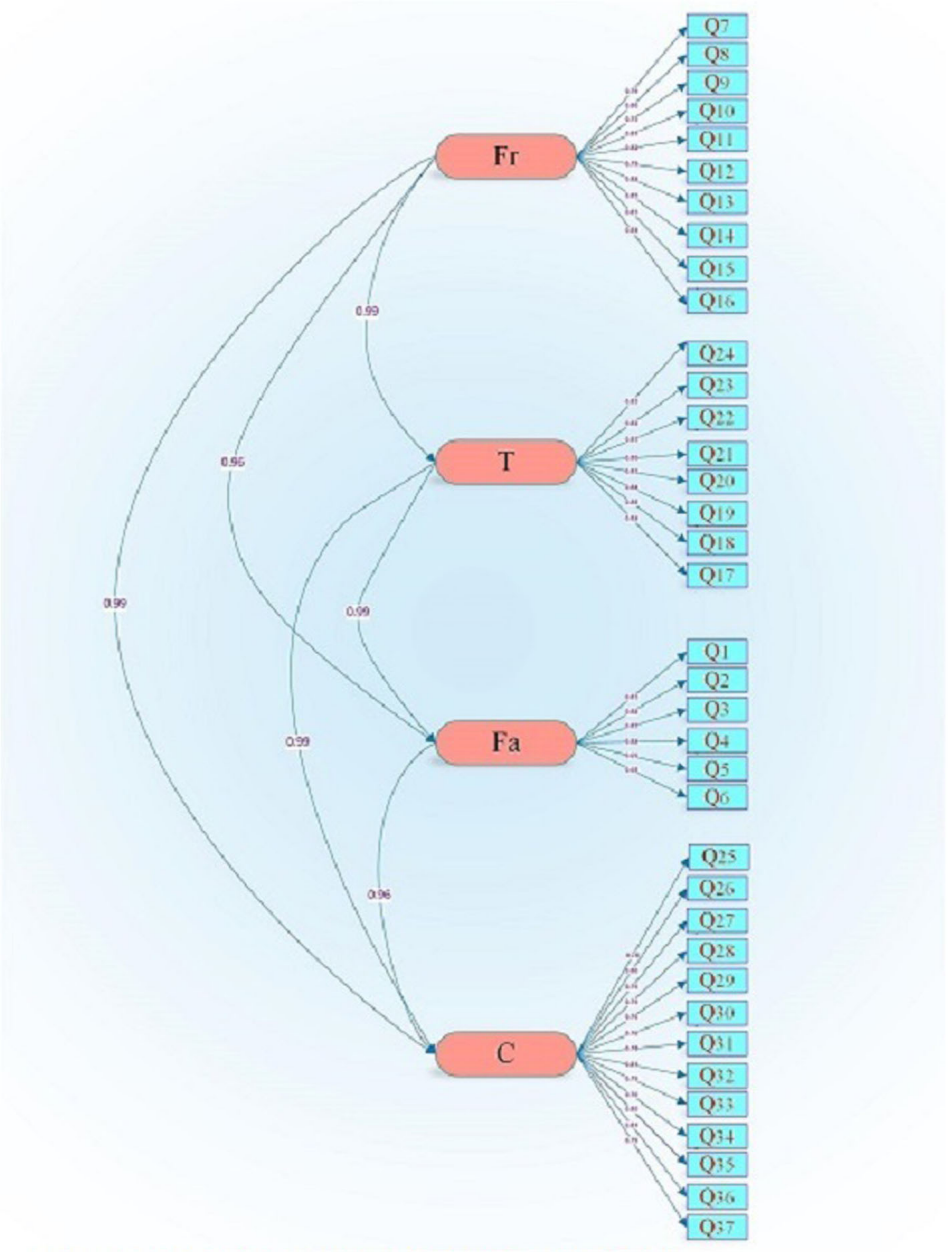
Four factor model of CVI in Iranian PWUDs

Figure 2 illustrates CFA model of the variable under study in standard mode and without coefficients. Since t-values in all cases are higher than |1.96| and the factor load is higher than 0.3 (Table 2), none of the statements were omitted. Based on the goodness of fit indices in CFA model, the goodness of fit of model with the collected data was supported (Table 3) (Fig. 2).

**Table 3:** Fit Indicators Confirmatory Factor Analysis Persian Version of CAI

Fit Indicators	Criterion	Level	Interpretation
χ^2^/DF	≥ 3	2.98	Optimal fit
CFI	>0.9	0.91	Optimal fit
NFI	>0.9	0.905	Optimal fit
GFI	>0.9	0.92	Optimal fit
TLI	>0.9	0.905	Optimal fit
RMSEA	<0.08	0.07	Optimal fit
R^2^	Near to 1	0.99	Optimal fit

With R2 = 0.99, 99% of variation of the dependent variable (total score of CAI) can be attributed to the independent variable (37 statements). In other words, 99% of changes in depended variable is caused by the independent variable.

With the support of normality of data in CAI items for PWUDs under methadone therapy, Pearson’s correlation test showed a direct and significant correlation at 99% confidence level (Table 4). In addition, there was is a direct and significant relationship between CAI subscales and total score of the tool (Table 5).

**Table 4:** Pearson correlation of items of CVI in Iranian PWUD

No	Items	Pearson correlation	
		R	*P*-value
1	My partner (the people I live with) and I are really a team in getting my life together	0.824	0.001^**^
2	When the going gets tough you are on your own	0.805	0.017^**^
3	My partner (the people I live with) knows what it takes to stay clean in my neighborhood	0.834	0.001^**^
4	My partner (the people I live with understand) understands what drug abuse does to a person	0.809	0.001^**^
5	My partner (the people I live with are) is pretty well informed about how treatment works	0.681	0.001^**^
6	I can tell my partner (the people I live with) everything	0.678	0.001^**^
7	Anytime I need something I can count on my family to help	0.789	0.001^**^
8	It is hard to talk to people in my family about my problems	0.801	0.001^**^
9	My family takes a big interest in how I am doing in treatment	0.786	0.001^**^
10	My family doesn’t know much about my life	0.804	0.001^**^
11	I’m part of a close family	0.826	0.001^**^
12	I can tell my family anything	0.798	0.001^**^
13	People in my family don’t really understand what drugs can do a person	0.823	0.001^**^
14	My family is standing by me throughout treatment	0.843	0.001^**^
15	People in my family pretty much know how treatment works	0.829	0.001^**^
16	My family doesn’t trust me	0.833	0.001^**^
17	Most of the people I hang out with like to keep their problems to themselves	0.822	0.001^**^
18	I have at least one friend I can count on to be there for me no matter what	0.84	0.001^**^
19	My friends support my efforts to turn my life around	0.839	0.001^**^
20	My friends can’t really understand my situation	0.83	0.001^**^
21	My friends ask me how my treatment is going	0.803	0.001^**^
22	I can’t really count on my friends to help me stay clean and out of trouble	0.826	0.001^**^
23	My friends know pretty well how treatment works	0.819	0.001^**^
24	I expect to have the same friends a year from now that I have today	0.836	0.001^**^
25	If I ever needed one, it would take an ambulance forever to get where I live	0.783	0.001^**^
26	There are good recreational programs in my neighborhood	0.8	0.001^**^
27	My neighborhood is full of drugs	0.788	0.001^**^
28	People in my neighborhood care about each other	0.795	0.001^**^
29	City services are a joke in my neighborhood	0.769	0.001^**^
30	I would say my neighborhood is a low crime area	0.733	0.001^**^
31	People in my neighborhood don’t believe treatment can do much	0.79	0.001^**^
32	It’s tough to stay out of trouble in my neighborhood	0.811	0.001^**^
33	Most of the people who live in my neighborhood are strongly anti-drug	0.786	0.001^**^
34	You have to watch your back in my neighborhood	0.79	0.001^**^
35	A lot of people in my neighborhood are working to stamp out drugs	0.797	0.001^**^
36	There’s not much for young people to do in my neighborhood	0.806	0.001^**^
37	Religion is strong in my neighborhood	0.797	0.001^**^

**P_value_ = < 0.001

**Table 5:** The internal reliability of CVI and dimensions among the subjects

Aspects of Community Assessment Inventory	Alpha Cronbach Coefficient
Support by the spouse/family with whom you are living (**Household**)	0.84
Support by the family outside the house (**Family**)	0.81
Support by friends (**Friends**)	0.8
Support by community (**Community**)	0.95
Total (Community Assessment Inventory)	0.85

To examine internal reliability of CAI, Cronbach’s alpha was computed for the whole tool equal to 0.85. Based on the results, the Farsi version of CAI had an acceptable internal consistency and the coefficient for the subscales of CAI ranged from 0.80 to 0.95 (Table 6).

**Table 6:** The correlation coefficient of CVI and dimensions with others in Iranian PWUD

Aspects CAI	Household (6 Items)	Family (10 Items)	Friends (8 Items)	Community (13 Items)	CAI
Household (6 Items)	1				
Family (10 Items)	0.887 0.001^*^	1			
Friends (8 Items)	0.922 0.001^*^	0.937 0.001^*^	1		
Community (13 Items)	0.891 0.001^*^	0.939 0.001^*^	0.937 0.001^*^	1	
Total (CAI)	0.941 0.001^*^	0.975 0.001^*^	0.976 0.001^*^	0.98 0.001^*^	1

*P_value_ = < 0.001

## Discussion

The CAI was translated and validated for PWUDs under methadone therapy in Iran. One feature of the study is that along with using the care-seekers’ opinions about face validity, the experts were also consulted about content validity. Through this, clarity and understandability of the statements were determined. Brown et al. (2004) examined face and content validity using the same method [13].

Construct validity of CAI in the subjects was measured using EFA and CFA. Halamova et al. (2018) followed the same approach [16] and Canty-Mitchel et al. (2000) used CFA to measure construct validity [17]. Clearly, factor analysis methods are commonly used for this purpose.

Factor analysis results supported all the 37 items and goodness of fit indices all were higher than 0.9, which supported goodness of fit of the tool (χ2/DF = 2.98, CFI = 0.91, NFI, TLI = 0.905 GF = 0.92, REMSEA = 0.07, R2 = 0.99). Halamova et al.(2018) also reported that goodness fit indices were above 0.9 (CFI = 0.98, NFI, TLI = 0.984 REMSEA = 0.045) and supported goodness of fit of the tool [16].

Pearson correlation coefficient also supported a significant and direct correlation of CAI subscales with each other and the total score of the tool. Brown et al. (2004) reported a direct and significant correlation between the subscales and the total score of the tool [13]. Canty-Mitchel et al. also reported similar results [17]. To elaborate on the findings, along with validating psychometric characteristics of the tool, consistent with other studies, our results also supported internal consistency of the scale.

As the results showed, the Farsi version of CAI has an acceptable internal consistency for PWUDs in Iran. Reliability of the subscales, based on Cronbach’s alpha was at 0.8–0.95 range. Therefore, the subscales are reliable. Khuong et al.(2018) also reported that Cronbach’s alpha of the tool was less than 0.81 [18]. A similar study by Priede et al. reported Cronbach’s alpha more than 0.7 [19]. Although, the number of subjects in these studies is different, the similar values of Cronbach’s alpha support the findings of the present study.

Data gathering was done using the questionnaire; therefore, it was not possible to examine subjective data. This, however, is a common feature of descriptive and tool validation works. Overcrowded clinics, boredom, muscles and joints pains, and lack of enough time to answer the items of the tool were some of the limitations of the study. These limitations prevented giving accurate answers to the questions in some of the subjects. To solve this problem, the author tried to explain the study design and its necessity and remove ambiguities if any.

## Conclusion

As the results showed, CAI had acceptable indices for Iranian PWUDs under methadone therapy. The tool can be used for assessing social support level in the study population. It is a reliable and valid tool for studies in pertinent fields. In general, CAI was a standard and acceptable tool for PWUD_s_ (Natural and industrial drugs) in Iran and future studies can use the tool.

## Data Availability

The datasets used and analyzed during the current study are available from the corresponding author on reasonable request.

## Abbreviations

CAI: Community Assessment Inventory
PWUD: Person/s with use/s drug/s
CVI: Content Validity Index
CVR: Content Validity Ratio
KMO: Kaiser Meyer Olkin
EFA: Explorative factor analysis
TLI: Tucker-Lewis Index
NFI: Normed Fit Index
GFI: Goodness of Fit Index
CFA: Confirmatory Factor Analysis
PC: Principle Components RMSEA Root Mean Square Error of Approximation
KUMS: Kermanshah University of Medical Sciences
